# Enhancing risk stratification models in localized prostate cancer by novel validated tissue biomarkers

**DOI:** 10.1038/s41391-024-00918-9

**Published:** 2024-11-14

**Authors:** Csilla Olah, Fabian Mairinger, Michael Wessolly, Steven Joniau, Martin Spahn, Marianna Kruithof-de Julio, Boris Hadaschik, Aron Soós, Péter Nyirády, Balázs Győrffy, Henning Reis, Tibor Szarvas

**Affiliations:** 1 Department of Urology, University of Duisburg-Essen, Essen, Germany; 2 Institute of Pathology, University Medicine Essen, University of Duisburg-Essen, Essen, Germany; 3 Department of Urology, University Hospitals Leuven, Leuven, Belgium; 4 Lindenhofspital, Bern, Switzerland; 5 Urology Research Laboratory, Department for BioMedical Research, University of Bern, Bern, Switzerland; 6 Department of Urology, Inselspital, Bern University Hospital, University of Bern, Bern, Switzerland; 7 Department of Urology, Semmelweis University, Budapest, Hungary; 8 Research Centre for Natural Sciences, Cancer Biomarker Research Group, Institute of Enzymology, Budapest, Hungary; 9 Department of Bioinformatics, Semmelweis University, Budapest, Hungary; 10 Dr. Senckenberg Institute of Pathology, University Hospital Frankfurt, Goethe University Frankfurt, Frankfurt am Main, Germany

**Keywords:** Prognostic markers, Cancer, Prostate cancer

## Abstract

**Background:**

Localized prostate cancer (PCa) is a largely heterogeneous disease regarding its clinical behavior. Current risk stratification relies on clinicopathological parameters and distinguishing between indolent and aggressive cases remains challenging. To improve risk stratification, we aimed to identify new prognostic markers for PCa.

**Methods:**

We performed an in silico analysis on publicly available PCa transcriptome datasets. The top 20 prognostic genes were assessed in PCa tissue samples of our institutional cohort (*n* = 92) using the NanoString nCounter technology. The three most promising candidates were further assessed by immunohistochemistry (IHC) in an institutional (*n* = 121) and an independent validation cohort from the EMPACT consortium (*n* = 199). Cancer-specific survival (CSS) and progression-free survival (PFS) were used as endpoints.

**Results:**

Our in silico analysis identified 113 prognostic genes. The prognostic values of seven of the top 20 genes were confirmed in our institutional radical prostatectomy (RPE) cohort. Low *CENPO, P2RX5*, *ABCC5* as well as high *ASF1B, NCAPH, UBE2C*, and *ZWINT* gene expressions were associated with shorter CSS. IHC analysis confirmed the significant associations between NCAPH and UBE2C staining and worse CSS. In the external validation cohort, higher NCAPH and ZWINT protein expressions were associated with shorter PFS. The combination of the newly identified tissue protein markers improved standard risk stratification models, such as D’Amico, CAPRA, and Cambridge prognostic groups.

**Conclusions:**

We identified and validated high tissue levels of NCAPH, UBE2C, and ZWINT as novel prognostic risk factors in clinically localized PCa patients. The use of these markers can improve routinely used risk estimation models.

## Introduction

Prostate cancer (PCa) is the most common cancer in males, with over 1.2 million new cases yearly worldwide, ranking fifth in cancer-related deaths [1]. The use of serum prostate-specific antigen (PSA) for early detection of PCa has resulted in a shift toward earlier diagnosis. However, it led to the detection of a high number of indolent PCa that do not necessitate active treatment and may remain asymptomatic throughout patient’s life [2]. Approximately 30–45% of PCa patients detected through screening are overdiagnosed, leading to unnecessary treatments like radical prostatectomy (RPE) or radiation therapy (RT) [3]. To avoid the risk of overtreatment, current guidelines do not recommend wide-spread population-based PSA screening, though debate persists [4]. Improved risk stratification is crucial for accurately distinguishing between low- and high-risk PCa cases, guiding treatment decisions towards active surveillance or treatment [5].

The D’Amico risk stratification - based on PSA level, clinical stage, and biopsy Gleason score - stratifies patients into low-, intermediate-, and high-risk groups. The original, retrospective study revealed that patients with intermediate- and high-risk PCa benefit more from RPE or RT regarding 5-year biochemical recurrence (BCR)-free survival compared to implant with or without neoadjuvant androgen deprivation therapy. On the other hand, patients with low-risk PCa showed no significant differences in response to various therapies [6]. The prognostic relevance of D’Amico classification for the prediction of BCR, progression, overall-, and cancer-specific survival was subsequently confirmed in patients who underwent RPE [7]. More recently, the Cambridge Prognostic Groups (CPG), a five-strata model utilizing PSA at diagnosis, clinical stage, and biopsy Gleason score, demonstrated superior predictive capability for PCa-related death compared to the previously described three-strata model [8]. Additional relevant risk estimation model, the Cancer of the Prostate Risk Assessment (CAPRA) scoring system, has been developed with the purpose to predict recurrence after RPE [9]. Each described model provides accuracies ranging from 75 to 90% for the prediction of adverse outcomes, however, did not take into account the tumor’s biology. In addition, gene panel-based classifiers were developed and validated earlier, however the selection of the included genes was performed using single mRNA datasets and thus may be limited. Additionally, the assay platform requirements hinder the widespread, decentralized application of these gene panels, while simple immunohistochemical analyses could be easily integrated into daily clinical routine.

In the last years, large, transcriptome datasets with detailed clinical annotation and the necessary bioinformatics tools have become accessible for PCa, enabling systematic search for prognostic biomarkers [10]. In the present work, we analyzed PCa transcriptome datasets [11, 12] to identify novel genes for the prediction of outcomes of clinically localized PCa after RPE. The top 20 prognostic genes were further assessed at the gene expression (GE) level in our institutional PCa cohort. Subsequently, the three most promising novel prognostic genes were further assessed at the protein level in two independent PCa cohorts.

## Materials and methods

### Overview of the analysis pipeline and patient cohorts

**Fig. 1:**
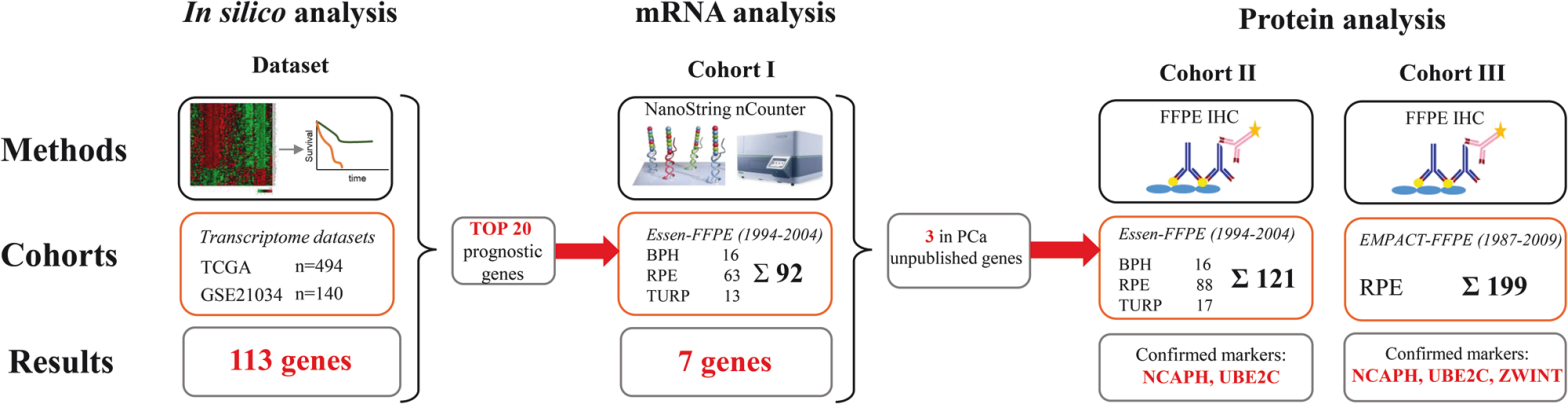
Overview of the performed analyses, patient cohorts and key findings of the present study.

Figure 1 provides an overview of the analysis steps, methods applied, and patient cohorts. Details of the in silico data analysis and description of the two institutional patient cohorts and external validation cohort are described in the supplementary materials.

Cohort I for GE analyses included 92 PCa patients who underwent either RPE (*n* = 63) or palliative transurethral resection (pTURP, *n* = 13) at the Department of Urology, University Hospital Essen as well as 16 men with benign prostatic hyperplasia (BPH). Cohort II for immunohistochemistry (IHC) analyses had a large overlap with cohort I and consisted of 121 men (RPE *n* = 88, pTURP *n* = 17, BPH *n* = 16). Cohort III included 199 intermediate and high-risk PCa patients who underwent RPE in eight European centers [13].

To gain a broader understanding of the expression patterns of selected candidate markers in various disease stages, we included not only clinically localized (RPE-treated) PCa patients but also samples from nonmalignant cases (BPH) and advanced PCa cases (pTURP). The results of these subgroups (BPH, pTURP) were only considered for comparison with clinically localized PCa but were not used for survival analyses. All analyses were performed blinded to the clinical data to minimize bias.

### GE analysis

Total RNA was extracted from FFPE sections with >40% tumor cell content as described in the supplementary materials.

### Immunohistochemistry

Based on the results of GE analyses, three protein markers (NCAPH Cat.Nr.: HPA003008, Sigma-Aldrich, St. Louis, Missouri, USA; UBE2C Cat.Nr.: H00011065-M01, Abnova, Taipei City, Taiwan; and ZWINT Cat.Nr.: ab84367, Abcam, Cambridge, Great-Britain) were selected for validation. The detailed description of IHC staining and evaluation is provided in the supplementary material.

### Statistical analyses

Ordinal clinicopathological characteristics were compared using nonparametric, two-tailed Wilcoxon rank-sum test. The relation between gene and protein expressions and ordinal variables with more than two categories were evaluated using Kruskal–Wallis test. Metric or continuous associations between protein/gene expressions and clinicopathological parameters were analyzed using Pearson chi-squared test and Spearman’s rank correlation coefficient. Cancer-specific and progression-free survival (CSS and PFS) analyses were performed using Cox proportional hazards analysis and visualized using the Kaplan–Meier curves with log-rank test. Parameters with significant values in the univariate analyses were further assessed in multivariate Cox models. In all analyses, *p*-value ≤ 0.05 was considered statistically significant. For evaluations of institutional cohorts, the SPSS software packages (IBM SPSS Statistics for Windows, version 25, IBM Corp., Armonk, NY, USA) and R Studio were used.

## Results

### Patients’ characteristics

The median follow-up for the RPE treated patients in cohort I, II, III were 179, 182 and 77 months, respectively. The median preoperative PSA values in the cohort I, II and III were 11.5, 10.8 and 36.8 ng/ml, respectively, which reflects the composition of cohorts; cohort I and II included low-, intermediate- and high-risk patients, while cohort III included only intermediate- and high-risk patients. Accordingly, both the clinical and pathological stage as well as lymph node (LN) positivity rate were less favorable in cohort III. The 5-year CSS rate was 87%, 90% and 94% for cohort I, II and III, respectively (Table 1).

**Table 1:** Patients’ characteristics for cohort I (for GE analysis) and cohorts II and III (for protein expression analysis).

Cohorts		Institutional GE cohort (I)			Institutional IHC cohort (II)			Multicentre IHC cohort (III)
		Whole cohort	pTURP	RPE	Whole cohort	pTURP	RPE	RPE
		*n* (%)	*n* (%)	*n* (%)	*n* (%)	*n* (%)	*n* (%)	*n* (%)
Patients		76	13	63	105	17	88	199
Age median [range]		64 [51–87]	67 [60–87]	62 [51–74]	63 [50–87]	69 [57–87]	62 [50–74]	67 [43–81]
PSA median [range]		13 [0–341]	39 [4–341]	11.5 [0–86]	11.3 [0–341]	62.6 [3–6–341]	10.8 [0–86]	36.8 [20–597]
cT stage	cT1	3 (4)	1 (8)	2 (3)	6 (6)	1 (6)	5 (6)	18 (9)
	cT2	46 (60)	5 (38)	41 (65)	61 (58)	4 (24)	57 (65)	79 (40)
	cT3	21 (28)	4 (31)	17 (27)	25 (24)	4 (24)	21 (24)	99 (50)
	cT4	4 (5)	2 (15)	2 (3)	5 (5)	2 (12)	3 (3)	3 (2)
	na	2	1	1 (2)	8	6	2 (2)	0
	T1	2 (3)	0	2 (3)	3 (3)	1 (6)	2 (2)	0 (0)
	pT2	36 (47)	5 (38)	31 (49)	49 (47)	3 (18)	46 (52)	33 (17)
	pT3	35 (46)	6 (46)	29 (46)	42 (40)	6 (35)	36 (41)	130 (65)
	pT4	3 (4)	2 (15)	1 (2)	6 (6)	3 (18)	3 (3)	36 (18)
	na	0	0	0	5	4	1	0
PostOP ISUP/WHO grade group	1	31 (41)	0	31 (49)	48 (46)	0	48 (55)	na
	2	16 (21)	0	16 (25)	22 (21)	0	22 (25)	na
	3	5 (7)	0	5 (8)	5 (5)	0	5 (6)	na
	4	11 (14)	3 (23)	8 (13)	9 (9)	2 (12)	7 (8)	na
	5	12 (16)	9 (69)	3 (5)	13 (13)	9 (53)	4 (4)	na
	na	1	1	0	8	6	2	199
LN metastasis (>2 cm)		8 (10)	3 (23)	5 (8)	10 (9)	3 (18)	7 (8)	77 (39)
D’Amico risk	low	5 (7)	–	5 (8)	10 (9)	–	10 (11)	0
	interm.	19 (26)	–	19 (30)	26 (24)	–	26 (30)	120 (60)
	high	51 (67)	12 (92)	39 (62)	63 (60)	13 (76)	50 (57)	79 (40)
	na	1	1	0	6	4	2	0
Capra risk	low	9 (12)	–	9 (14)	21 (20)	–	21 (24)	na
	interm.	33 (43)	–	33 (52)	37 (35)	–	37 (42)	na
	high	31 (55)	11 (85)	20 (32)	37 (35)	11 (65)	26 (30)	na
	na	3	2	1	10	6	4	199
Death		59 (78)	12 (92)	47 (75)	83 (79)	15 (88)	68 (77)	30 (15)
Cancer-spec. death		29 (38)	11 (85)	18 (29)	37 (35)	14 (82)	23 (26)	13 (7)
Median follow-up in months	[range]	151 [8–340]	43 [1–151]	179 [1–340]	156 [1–340]	18 [1–99]	182 [1–340]	77 [1–154]
Progression		na	na	na	na	na	na	57 (29)
PSA-progression		na	na	na	na	na	na	56 (28)

*LN* lymph node metastasis, *interm* intermediate, *na* no available data, *RPE* radical prostatectomy, *pTURP* palliative transurethral resection, *GE* gene expression.

### GE analysis of 20 genes in the institutional GE cohort (cohort I)

Our in silico analysis identified 113 prognostic genes in TCGA and GSE21034 datasets (Supplementary Table 1). The top 20 of 113 genes with the highest average hazard ratio values and with the lowest p-values were selected. Then, we measured the GE levels of these genes in our institutional GE cohort (cohort I). GE as continuous variables were correlated with clinicopathological data (Supplementary Table 2).

**Fig. 2:**
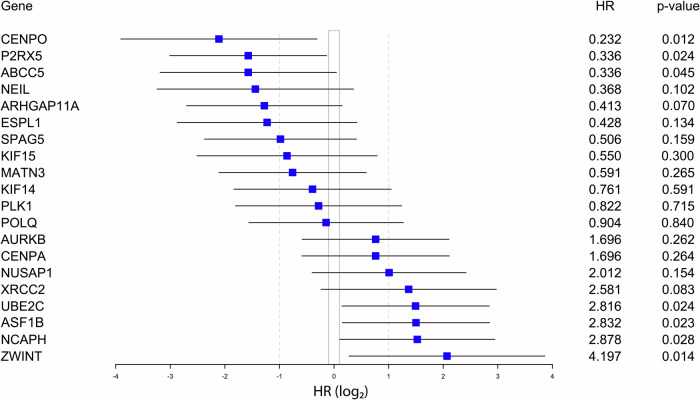
Forest plot presentation of Cox univariate CSS analyses for the 20 genes with dichotomized GE levels in the institutional GE cohort (cohort I). HR hazard ratio.

Seven of 20 genes showed significant prognostic value: high expression of *UBE2C, NCAPH, ASF1B*, and *ZWINT* (risk genes), and low expression of *CENPO, P2RX5*, and *ABCC5* (protective genes) were associated with shorter CSS after RPE (Fig. 2). For further investigation, we focused on those three risk markers which have formerly not been assessed in PCa (UBE2C, NCAPH, ZWINT).

**Fig. 3:**
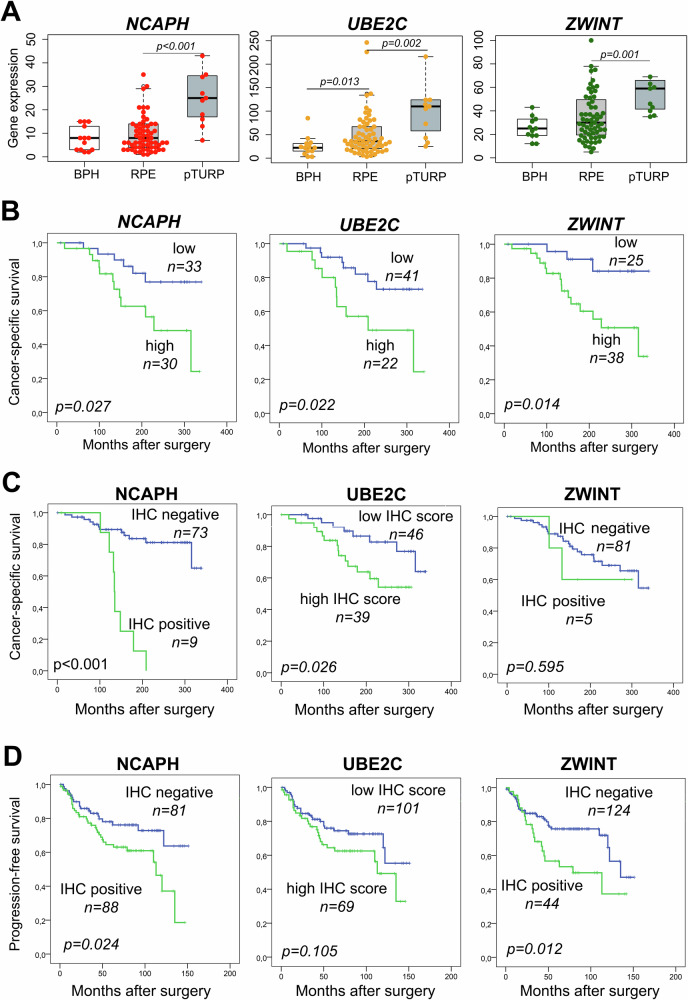
Gene expression (GE) values of selected risk genes in BPH, RPE and pTURP samples. GE levels of NCAPH, UBE2C and ZWINT were significantly higher in pTURP compared to BPH samples (**A**). Cancer-specific survival (CSS) analyses stratified by GE levels in the institutional GE cohort (cohort I) (**B**). CSS analyses stratified by protein expressions in the institutional IHC cohort (cohort II) (**C**). Progression-free survival (PFS) analyses of protein expressions in the multicentre validation cohort (cohort III) (**D**). BPH benign prostate hyperplasia, RPE radical prostatectomy, pTURP palliative transurethral resection of prostate.

These genes were significantly overexpressed in advanced PCa cases (pTURP) compared to both BPH and RPE groups. In addition, *UBE2C* showed significantly lower expression levels in BPH samples compared to RPEs (Fig. 3A).

*NCAPH* and *ZWINT* GE showed no association with clinicopathological parameters, while higher *UBE2C* GE levels were associated with positive LNs (*p* = 0.022), higher ISUP/WHO grade (*p* = 0.013), and higher CAPRA score (*p* = 0.036) (Table 2).

**Table 2:** Association between patients’ clinicopathological parameters and NCAPH, UBE2C, and ZWINT gene and protein expressions in the institutional GE and IHC cohorts (I and II).

		Institutional GE cohort (cohort I)										Institutional IHC cohort (cohort II)																
Variables		*NCAPH*			*UBE2C*			*ZWINT*			NCAPH IHC score						UBE2C IHC score						ZWINT IHC score					
	*n*	Med	Range	*P*	Med	Range	*P*	Med	Range	*P*	*n*	0 *n*	%	≥1 *n*	%	*P*	*n*	≤1 *n*	%	>1 *n*	%	*P*	*n*	0 *n*	%	≥1 *n*	%	*P*
Age																												
≤65 years	38	6	1–92	0.784	36	3–226	0.452	29	5–349	0.415	52	47	90%	5	10%	0.566	55	26	47%	29	53%	0.058	55	51	93%	4	7%	0.461
>65 years	25	10	1–61		34	5–246		33	10–75		29	25	86%	4	14%		29	20	69%	9	31%		30	29	97%	1	3%	
Pathological stage																												
T1-pT2	33	6	1–61	0.291	37	5–137	0.880	32	9–214	0.262	44	41	93%	3	7%	0.180	46	26	57%	20	43%	0.721	46	43	93%	3	7%	0.786
pT3-pT4	30	8.5	1–92		35	3–226		28.5	5–349		37	31	84%	6	16%		38	20	53%	18	47%		39	37	95%	2	5%	
Lymph node status																												
LN0	58	7	1–92	0.079	34	3–246	0.022	29	5–349	0.461	74	66	89%	8	11%	0.780	77	45	58%	32	42%	0.025	78	73	94%	5	6%	0.490
LN+	5	17	3–29		73	49–229		43	17–59		7	6	86%	1	14%		7	1	14%	6	86%		7	7	100%	0	0%	
PSA																												
<10 ng/ml	24	7	2–59	0.496	28	5–134	0.065	29	10–100	0.800	36	36	100%	0	0%	0.004	40	29	73%	11	28%	0.003	39	36	92%	3	8%	0.530
≥10 ng/ml	38	8.5	1–92		38	3–246		32.5	5–349		44	35	80%	9	20%		43	17	40%	26	60%		45	43	96%	2	4%	
PSA as continuous (KW)	63	8	1–92	0.405	36	3–246	0.424	30	5–349	0.522	80					–	83					–	84					–
ISUP/WHO grade group																												
1	31	6	2–61	0.461	26	5–246	0.013	33	8–214	0.885	46	44	96%	2	4%	0.023	46	30	65%	16	35%	0.025	46	43	93%	3	7%	0.808
2–5	32	9	1–92		47	3–226		28.5	5–349		34	27	79%	7	21%		37	15	41%	22	59%		38	36	95%	2	5%	
ISUP continuous (KW)	63	8	1–92	0.365	36	3–246	0.073	30	5–349	0.403	80					–	83					–	84					
D'Amico																												
Risk group 1-2	24	6	1–61	0.542	32	3–137	0.232	32	5–214	0.944	35	31	89%	4	11%	0.964	34	20	59%	14	41%	0.483	34	30	88%	4	12%	0.063
Risk group 3	39	8	2–92		36	8–246		28.5	8–349		45	40	89%	5	11%		49	25	51%	24	49%		50	49	98%	1	2%	
D'Amico continuous (KW)	63	8.5	1–92	0.392	36	3–246	0.458	30	5–349	0.613	80					–	83					–	84					–
CAPRA																												
Risk group 1-2	42	7	1–92	0.402	31.5	3–246	0.036	32.5	5–349	0.470	56	51	91%	5	9%	0.282	55	34	62%	21	38%	0.049	57	53	93%	4	7%	0.573
Risk group 3	20	8.5	2–30		49.5	14–226		26.5	13–78		23	19	83%	4	17%		26	10	38%	16	62%		26	25	96%	1	4%	
CAPRA continuous (KW)	62	8	1–92	0.394	36	3–246	0.066	30	5–349	0.430	79					–	81					–	84					–

Bold printed *p*-values were significant (≤0.05).

*KW* Kruskal–Wallis test was performed for the continuous variables.

In the univariate analysis, high PSA (≥10 ng/ml, *p* = 0.009), continuous CAPRA score (*p* = 0.001), and high *NCAPH* (*p* = 0.034), *UBE2C* (*p* = 0.028) and *ZWINT* (*p* = 0.023) GE levels were associated with shorter CSS. In the multivariate analysis, only PSA remained independent significant risk-factor for CSS (*p* = 0.018) (Supplementary Table 3A, B).

### IHC results of UBE2C, NCAPH, ZWINT in the institutional IHC cohort (cohort II)

Positive NCAPH staining showed a significant association with higher PSA levels (*p* = 0.004) and ISUP/WHO grade (*p* = 0.023). Consistent with the observations at the GE level, higher UBE2C protein expression significantly associated with LN positivity (*p* = 0.025), and higher PSA level (*p* = 0.003), ISUP/WHO grade (*p* = 0.025), and CAPRA risk score (*p* = 0.049). Finally, neither ZWINT GE nor protein expression showed an association with any of the clinicopathological parameters (Table 2).

We compared GE and protein levels of the three genes in overlapping samples and observed a significant correlation between gene and protein expression levels for UBE2C (*p* = 0.015), but not for NCAPH and ZWINT (Supplementary Fig. 2).

Similar to the results found at the GE level, NCAPH and UBE2C protein expressions were significantly associated with CSS (*p* < 0.001, and *p* = 0.026, respectively). However, high ZWINT protein expression showed no association with CSS, possibly due to the fact that only 5 of 86 (6%) PCa cases exhibited positive ZWINT staining (Fig. 3B, C).

In addition to the markers, high PSA (*p* = 0.002), LN positivity (*p* = 0.029), high ISUP/WHO grade (*p* = 0.026), continuous CAPRA score (*p* = 0.017), and high D’Amico scores (*p* = 0.049) proved to be significantly associated with poor CSS (Supplementary Table 3A). In the multivariate analysis, only high PSA and NCAPH expression remained independent significant risk-factors for CSS (*p* = 0.023, *p* = 0.04) (Supplementary Table 3B).

### IHC results of UBE2C, NCAPH, ZWINT in the independent multicentre IHC cohort (cohort III)

Protein expressions of NCAPH, UBE2C and ZWINT were investigated in an independent multicentre validation cohort (cohort III) consisting of intermediate and high-risk PCa patients treated with RPE (*n* = 199). Patients with high NCAPH and ZWINT protein expressions showed significantly shorter PFS (*p* = 0.024, *p* = 0.012) (Fig. 3D). In cohort III, a substantially higher proportion of patients were tested positive for ZWINT (26%), compared to to 6% in cohort II, which may be explained by the higher rate of high-risk patients. In cohort III, PFS was used as an endpoint, since only 13 PCa-related deaths (6%) were registered largely limiting the statistical evaluability of CSS as an endpoint. Therefore, results for CSS are only detailed in the supplementary materials.

We found no relationship between the clinicopathological parameters and CSS or PFS, only high ZWINT for CSS (*p* = 0.041), and high NCAPH and ZWINT expressions showed a significant association with higher risk of progression after RPE (*p* = 0.04, *p* = 0.010). Both markers proved to be prognostic in the multivariate analysis (*p* = 0.002, *p* = 0.010) (Supplementary Table 3A, B).

### Evaluation of clinical risk groups with and without NCAPH, UBE2C or ZWINT expressions

According to current guidelines, patients are classified into low-, intermediate-, and high-risk groups based on clinicopathological variables. In our cohorts, the D’Amico and CAPRA risk scores adequately identified patients with low-risk PCa (100% 5- and 10-year CSS). In contrast, intermediate and high-risk groups had similar CSS or PFS rates showing a limited prognostic value for the above risk models. Most importantly, high-risk patients (according to both D’Amico and CAPRA) could be further stratified by NCAPH, UBE2C, and ZWINT gene and protein expressions into subgroups with significantly different prognosis. The one-by-one addition of NCAPH, UBE2C and ZWINT expressions to the established prognostic models are shown in Supplementary Figs. 3 and 4.

### Evaluation of clinical risk groups with and without the three protein IHC score

The combined protein expressions of NCAPH, UBE2C and ZWINT proved to be strong risk factors for patients’ outcome in both, the institutional and validation cohorts (cohort II and III) (Supplementary Table 4A, C). High expression of one of the three markers were associated with decreased CSS in cohort II and decreased PFS in cohort III (*p* = 0.006, *p* = 0.023).

**Fig. 4:**
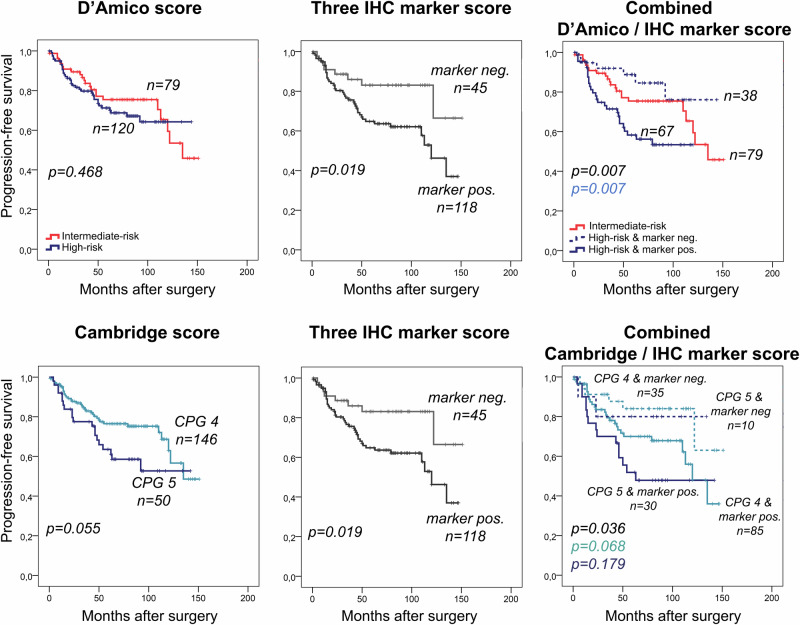
Progression-free survival (PFS) analysis by D’Amico and Cambridge risk scores (left plots), three IHC marker score (middle plots), and the combination of D’Amico or Cambridge with IHC marker scores (right plots) in the multicentre IHC validation cohort (cohort III). In the D’Amico high-risk and Cambridge prognostic groups (CPG 4 and 5) patients were further stratified by the three maker IHC marker score (at least one marker high). *P*-values represent PFS differences for all groups (black), and between D’Amico high-risk patients with negative vs. positive marker groups (blue), and between Cambridge risk group 4 (CPG) patients with negative vs. positive marker groups (light blue) and between CPG 5 patients with negative vs. positive marker groups (dark blue). The three IHC marker score evaluation was not feasible in 36 samples due to missing IHC staining of any of the three markers. Neg.: negative marker expression, Pos.: positive expression of any of the three marker.

The high protein expression of any of the three markers (three IHC marker score) significantly improved D’Amico risk stratification by splitting the high-risk patients into a favorable *vs*. a very poor prognosis subgroup in cohort III for PFS and CSS (*p* = 0.007 and *p* = 0.065). In addition, patients in the D’Amico high-risk group but favorable IHC results (all three markers negative) had even longer CSS and PFS than D’Amico intermediate risk-group patients (Fig. 4, Supplementary Fig. 5).

The five-tired Cambridge model (CPG 1–5) was superior to the three-tired D’Amico model regarding its prognostic value for both PFS and CSS in the external validation cohort (cohort III) (CSS: *p* = 0.247 *vs*. *p* < 0.001 (Supplementary Fig. 5) and PFS: *p* = 0.468 *vs*. *p* = 0.055 (Fig. 4)). The higher risk heterogeneity and lower number of patients in each risk group did not allow for the feasible application of the Cambridge model to the institutional cohort II. As the validation cohort (cohort III) included only intermediate and high-risk patients, only three patients were included in the CPG 3 according to the Cambridge model (not presented on the Figures), while 146 patients were classified into the CPG 4 and 50 in the CPG 5 groups. The CPG 5 subgroup showed significantly worse CSS (*p* < 0.001, Supplementary Fig. 5) and slightly shorter PFS (*p* = 0.055, Fig. 4) compared to CPG 4. We divided the two subgroups with the three IHC marker score (combination of NCAPH, UBE2C and ZWINT – at least one positive), and found no PCa-caused death in CPG high-risk [4, 5] but marker negative groups (Supplementary Fig. 5). When considering CSS endpoint, no PCa-caused death was observed in the marker negative subgroup (*p* = 0.023, Supplementary Fig. 5) and only 8 (18%) patients progressed after RPE (*p* = 0.019, Fig. 4).

## Discussion

In the present work, we demonstrate, for the first time, that high gene and protein expressions of NCAPH, UBE2C, and ZWINT are associated with poor prognosis in clinically localized PCa. Furthermore, the addition of GE or protein expression of these markers to established clinical risk scores substantially increased their prognostic value, especially in the high-risk groups.

PCa exhibits diverse molecular and histological patterns, leading to a wide range of clinical presentations. While current prognostic classifications combine PSA level, clinical stage and biopsy Gleason score, they do not take into account the tumor’s molecular characteristics. Recent advancements in molecular techniques have facilitated a comprehensive exploration of the molecular underpinnings of PCa, which led to the description of distinct molecular subtypes on the DNA and mRNA levels. However, the clinical application of subtype classification remains experimental and awaits further validation [14].

Further efforts aimed to incorporate molecular GE signatures into routine prognostication. The 31-gene panel *Prolaris* was shown to predict BCR after RPE [15]. The 22-gene *Decipher* classifier was found to predict metastatic progression after RPE [16]. Although these tools are recommended by the current *NCCN* guidelines, they are not yet widely adopted in the routine [17, 18].

Since the elaboration of the above GE-tools, larger independent transcriptome datasets became available to identify novel, easy-to-use prognostic biomarkers. We performed a systematic in silico analysis and identified 113 prognostic genes. GE of the 20 most significant genes were determined in our institutional RPE cohort. Of note, our top 20 genes showed some overlapping with the previously published commercially available gene panels. For instance, the *Prolaris* panel included the *NUSAP1, PLK1*, and *ASF1B* [15], while the *Decipher* panel includes *NUSAP1* and *UBE2C* [16]. In the first validation step, we confirmed the prognostic values of 7 of the top 20 genes; low GEs of *CENPO, P2RX5* and *ABCC5* and high GEs of *ASF1B, NCAPH, UBE2C*, and *ZWINT* showed significant associations with shorter CSS in our RPE cohort (cohort I). Consequently, we selected *NCAPH, UBE2C*, and *ZWINT* for further validation on the protein level in our institutional cohort and an independent multicentre cohort.

NCAPH regulates mitotic chromosome architecture and segregation, impacting cell growth, proliferation, and migration. Inhibition in non-small cell lung cancer cells arrested cell cycle at the G2/M phase, leading to apoptosis [19], and its high expression was associated with advanced stage and poor survival [20]. In cervical cancer, NCAPH is significantly upregulated, correlating with tumor size, invasion, and LN metastasis [21]. In the TCGA PCa dataset, *NCAPH* is significantly overexpressed in tumors, associated with advanced stage, LN involvement, and worse OS [22]. Silencing *NCAPH* expression inhibited proliferation and migration in PCa cell lines [23]. Accordingly, in our study, higher gene and protein expressions were associated with worse CSS and PFS in localized PCa. High *NCAPH* GE tended to associate with the presence of LN metastasis, while higher protein expression was significantly associated with higher PSA level, and ISUP/WHO grade.

UBE2C is involved in cell cycle regulation. Increased UBE2C expression is associated with larger tumor size, higher grade, lymphovascular invasion, LN metastasis, and poor survival in various tumors [24–27]. In PCa, in silico analyses revealed that high *UBE2C* GE is associated with higher Gleason scores and worse survival [28]. In a recent study, UBE2C was associated with higher PSA, Gleason score and pathological stage but no associations were found with outcomes [29]. In accordance, we observed that high gene and protein expressions are associated with higher PSA levels, ISUP/WHO grade, LN positivity and poor CSS.

ZWINT is a component of the kinetochore complex, critical for cell division. Little is known about the role and prognostic value ZWINT in various malignancies. In NSCLC, higher *ZWINT* GE was observed in tumors compared to non-cancerous tissues. High GE was associated with significantly worse OS and PFS in patients with pulmonary adenocarcinoma [30]. In the present study, *ZWINT* GE was significantly higher in advanced PCa cases (pTURP) and higher ZWINT levels were associated with shorter CSS and PFS.

When applying established clinicopathological risk scores like D’Amico and CAPRA to our cohorts, we found that they accurately identify low-risk patients with excellent prognosis. However, CSS and PFS between intermediate and high-risk patients were not significantly different with either risk classifications. Combining the three molecular markers with the D’Amico risk score revealed prognostic value specifically in high-risk patients, suggesting heterogeneity within this group. D’Amico high-risk patients with low NCAPH, UBE2C and ZWINT levels have significant better prognosis compared to those with high expression levels. Similarly, for the CAPRA model, our markers were able to split high-risk patients in two significantly different prognostic groups. We could reach an even better prognostic stratification when combining the three protein markers (at least one highly expressed). The five-tired Cambridge risk model was superior compared to the three-tired D’Amico model in terms of CSS and PFS. The combined use of IHC markers (all three markers negative) could identify patients with improved survival in the CPG 4-5 subgroups. More importantly, no cancer-specific death was detected in the three marker negative subgroup. Based on these, we concluded that our newly identified and validated markers can improve currently used risk models independent of using the markers on the GE or protein levels. Therefore, a straightforward IHC staining of three markers could identify patients with excellent prognosis after RPE.

Our study is limited by its inherent retrospective nature, which may introduce uncontrolled selection bias and is associated with missing data points (marked as ‘NA’ in separate rows of Table 1). Additionally, there is variability in the availability of some endpoints; for example, PFS data was available only for cohort III, while CSS data was available for cohorts I and II but limited by the shorter follow-up time in cohort III. Finally, the number of patients with available mRNA samples was limited.

In our study, using a systematic hypothesis-free approach, we identified and validated – for the first time - UBE2C, NCAPH, and ZWINT as novel prognostic tissue markers - for localized PCa. Combining these biomarkers with established prognostic scores, we were able to substantially improve risk stratification especially within the high-risk group.

## Supplementary information

Enhancing Risk Stratification models in Localized Prostate Cancer by Novel Validated Tissue Biomarkers

## Data Availability

Data sources and handling of the publicly available datasets used in this study are described in the Materials and Methods. Further details and other data that support the findings of this study are available from the corresponding authors upon request.
