# Supplementary material for: Enhancing risk stratification models in localized prostate cancer by novel validated tissue biomarkers
Source: Prostate Cancer Prostatic Dis. 2024 Nov 14;28(3):773–81. doi: 10.1038/s41391-024-00918-9 (PMC12399428; doi:10.1038/s41391-024-00918-9)
Supplement: Supplementary file 1 — Enhancing Risk Stratification models in Localized Prostate Cancer by Novel Validated Tissue Biomarkers [file 41391_2024_918_MOESM1_ESM.docx]

**Enhancing Risk Stratification models in Localized Prostate Cancer by Novel Validated Tissue Biomarkers**

*Csilla Olah, Fabian Mairinger, Michael Wessolly, Steven Joniau, Martin Spahn, Marianna Kruithof-de Julio, Boris Hadaschik, Aron Soós, Péter Nyirády, Balázs Győrffy, Henning Reis, Tibor Szarvas*

# **Supplementary materials**

## *In silico data analysis*

Two independent transcriptome datasets form patients with clinically localized prostate cancer (PCa) and available follow-up data were downloaded from the cBioPortal (https://www.cbioportal.org/study/summary?id=prad_tcga_pan_can_atlas_2018) (TCGA, n=494) and Gene Expression Omnibus (GSE21034, n=140). For the TCGA dataset, overall survival (OS), while for the GSE21034 dataset, both OS and biochemical recurrence-free survival were used as endpoints. Genes with statistically significant associations with survival (p≤0.05) were ranked according to their average risk ratio values (HR), ​and the top 20 genes were selected for further analysis (Supplementary Table 1). In silico data analyses were performed by R Studio.

## *Patient cohorts*

### *Institutional GE cohort (cohort I; n=92)*

For gene expression (GE) analysis of the selected 20 candidate genes, formalin-fixed paraffin-embedded (FFPE) tissue samples were collected from 121 men who underwent surgical prostate treatment between 1994 and 2004 at the Department of Urology of the University Hospital Essen. Inclusion criteria required a tumor cell content of ≥40% in the available tissue sample and the availability of follow-up data. Twenty-nine patients were excluded due to low tumor cell content. Of the included 92 patients; 63 received RPE for clinically localized PCa, 13 patients with advanced PCa were treated with palliative transurethral resection (pTURP), and 16 patients with benign prostatic hyperplasia (BPH) had transurethral resection of the prostate (TURP).

To gain a broader understanding of the expression patterns of selected candidate markers in various disease stages, we included not only clinically localized (RPE-treated) PCa patients but also samples from nonmalignant cases (BPH) and advanced PCa cases (pTURP). The results of these subgroups (BPH, pTURP) were only considered for comparison with clinically localized PCa but were not used for survival analyses. Time-to-event evaluations (cancer-specific survival (CSS) and progression-free survival (PFS) analyses) were conducted only for RPE-treated (but not for pTURP and BPH) patients.

### *Institutional IHC cohort (cohort II; n=121)*

For protein expression analyses via immunohistochemistry (IHC) of three selected proteins, an FFPE cohort showing a large overlap with the GE cohort (cohort I) was prepared (RPE n=88, pTURP n=17, BPH n=16). For cohort II long-term CSS data is available as an endpoint.

### *Independent multicentre IHC cohort (cohort III; n=199)*

For further validation of the three selected protein markers, an independent multicentre validation cohort (cohort III) was used. This formerly published (EMPACT-study) validation cohort included 199 patients with intermediate and high-risk PCas, who underwent RPE between 1987 and 2009 at eight European tertiary centers (1). For cohort III, PFS was used as the primary endpoint. Progression was defined by either PSA-progression or clinical events after RPE. CSS was also available; however, this endpoint was limited by the low number of cancer-related deaths (n=13).

## *Patients’ risk stratification*

Patients were stratified into risk groups based on D’Amico, CAPRA, and Cambridge risk models (2-4). For the CAPRA model, the RPE tumor cell content was applied. CAPRA score calculation was not possible for the external validation cohort due to missing data on the percentage of biopsy core positivity.

## *Gene expression analysis and data normalization*

Total RNA was extracted from 5 μm FFPE tumor containing sections by using RNeasy DSP FFPE Kit (Qiagen, Hilden, Germany, Cat.Nr.: 73604) according to the manufacturer’s instructions. RNA concentration and quality were determined by a Multiskan GO microplate spectrophotometer (Thermo Scientific, Waltham, MA, USA Cat.Nr.: N12391). For the GE analysis, 100 ng total RNA was applied using the NanoString nCounter Analysis System (NanoString Technologies, Seattle, WA, USA). For data analysis, the nSolver 4.0 Software was applied. Count data of NanoString analysis were normalized using technical correction by subtracting the mean count data of the eight negative controls plus two-times standard deviation from all target count data. Next, GE levels were normalized to the geometric mean of two reference genes (*GAPDH, HPRT1*). Finally, all GE levels not exceeding p<0.05 after correlating count data of negative controls with two-times standard deviation of count data per gene were considered insufficient to overcome background noise. Expression level of each gene was correlated with clinicopathological and follow-up data.

## *Cut-off determination of the top genes*

For the gene expression analysis of the top 20 genes selected from in silico results (*ABCC5, AFS1B, ARHAGP11A, AURKB, CENPA, CENPO, ESPL1, KIF14, KIF15, MATN3, NCAPH, NEIL3, NUSAP1, P2RX5, PLK1, POLQ, SPAG5, UBE2C, XRCC2, ZWINT*) the best cut-off values were determined. Cut-off values of the top 20 genes were calculated according to the following process: first, the upper (Q3) and lower quartiles (Q1) of expression values of each gene were determined, then the best cut-off value between the upper and lower quartiles was identified by evaluating all possible cut-off values in the Cox regression analysis and finally computing the Benjamini-Hochberg False-Discovery Rate. Cut-off determination was performed by R Studio.

## *Immunohistochemistry*

IHC staining was performed on 5 μm thick sections of FFPE samples of both the institutional (cohort II) and the independent multicentre validation cohort (cohort III). All three antibodies were pre/treated at 95°C for 30 minutes with NCAPH and ZWINT at high and UBE2C at low pH. All antibody-reactions were developed with a dilution of 1:100 and incubated for 30 minutes each. A linker was used for NCAPH.

Immunohistochemical staining (IHC scores) of the three proteins was evaluated by uropathologist (H.R) based on staining intensities and scored as 0 (negative), 1 (weak), 2 (moderate), and 3 (strong). For UBE2C, samples with negative and weak staining intensities were merged into low IHC score, while samples with moderate and strong intensities were merged into high IHC score. For ZWINT and NCAPH, the samples were divided into negative (0) and positive groups (1, 2, 3).

NCAPH showed a cytoplasmic staining; UBE2C exhibited both nuclear and cytoplasmic staining, while ZWINT showed a nuclear localization (Supplementary Figure 1). Because of technical reasons, staining for six, three and two RPE cases of NCAPH, UBE2C, and ZWINT, respectively could not be evaluated in institutional IHC cohort (cohort II). While, staining for 30, 29 and 31 cases of NCAPH, UBE2C, and ZWINT could not be evaluated in independent multicentre IHC cohort (cohort III).

## *Supplementary Figures*

**
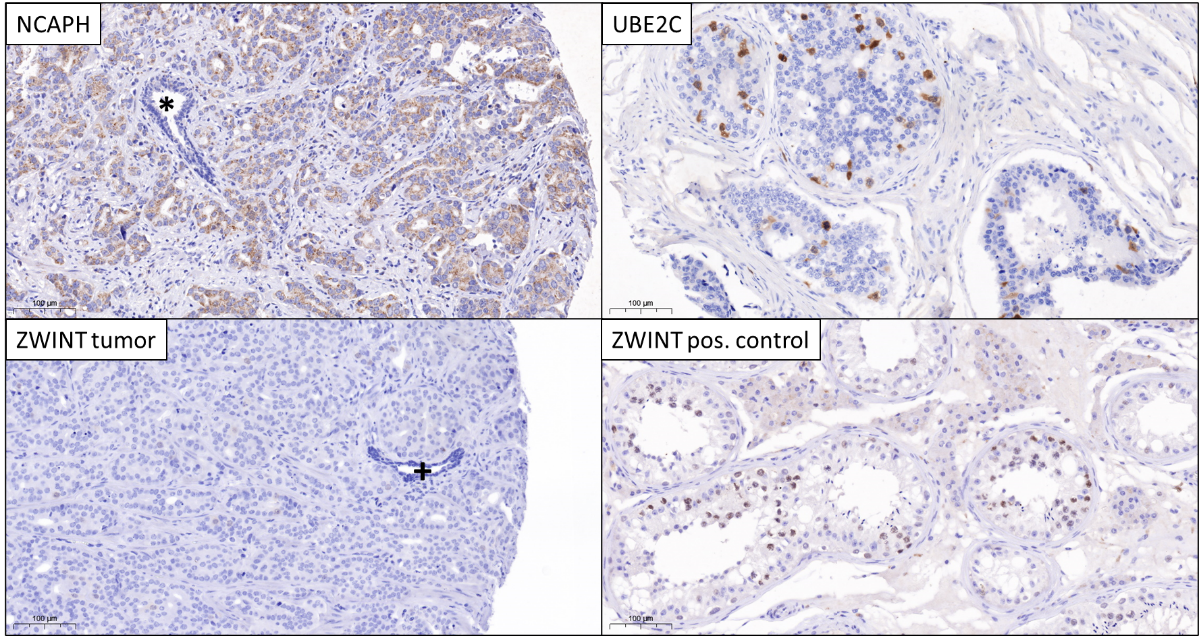
**

**Supplementary Figure 1**. Immunohistochemical staining of NCAPH, UBE2C, and ZWINT. NCAPH: Cytoplasmic staining is observed in tumor cells, but not in the normal gland (*). UBE2C: Moderate to strong cytoplasmic and nuclear staining is present in some tumor cells. ZWINT (tumor): Weak nuclear reactivity is observed in a few tumor cells but not in the benign gland (+). ZWINT (positive control): Positive control tissue shows moderate to strong nuclear reactivity in testicular germ cells.


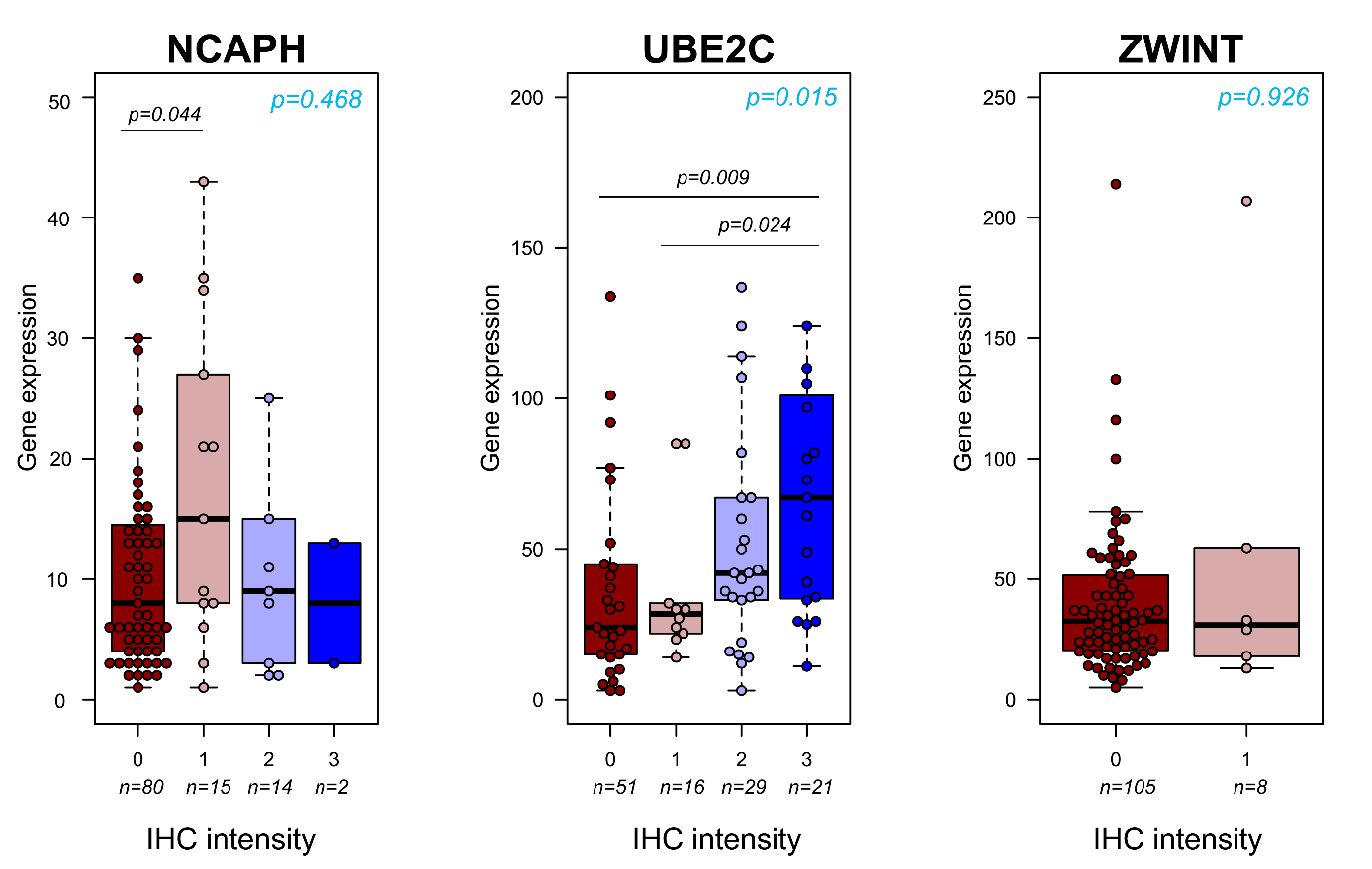


**Supplementary Figure 2.** Relationship between gene expression levels and IHC intensities for the three markers using Kruskal-Wallis test. P-values with blue represents the results of Kruskal-Wallis test. The gene expression differences between the groups with different IHC intensities (ranging from 0 to 3, or 0 to 1) are analyzed using Wilcoxon rank sum-test. P-values with black represents the results of Wilcoxon rank sum-test. IHC: immunohistochemical.


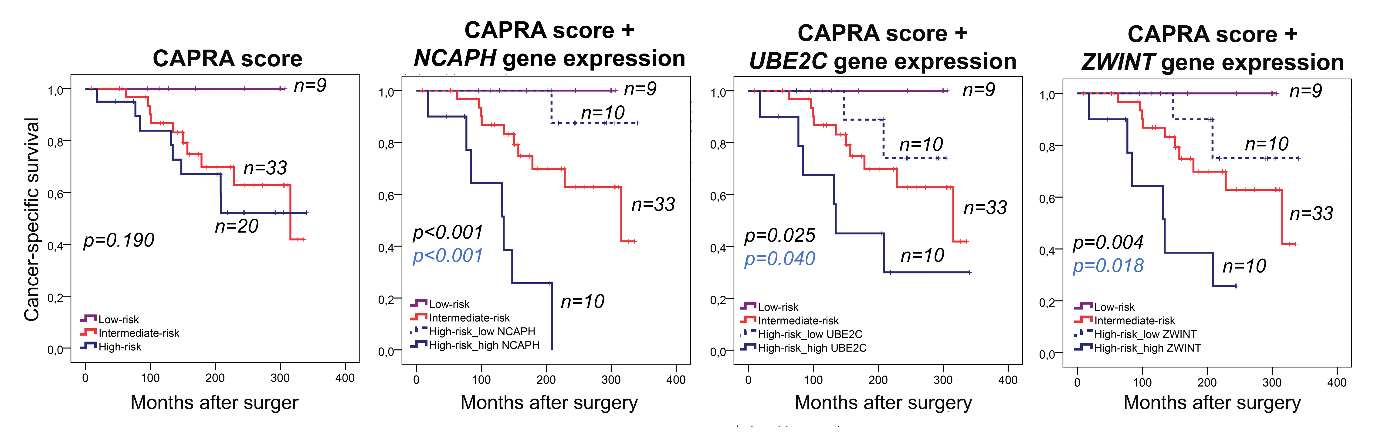


**Supplementary Figure 3**. Cancer-specific survival (CSS) analysis stratified by CAPRA risk scores in institutional GE cohort (cohort I). High-risk patients were further stratified by the gene expression of *NCAPH, UBE2C* and *ZWINT*. P-values represent CSS differences for all groups (black) and between high-risk patients with low vs. high gene or protein expressions (blue).


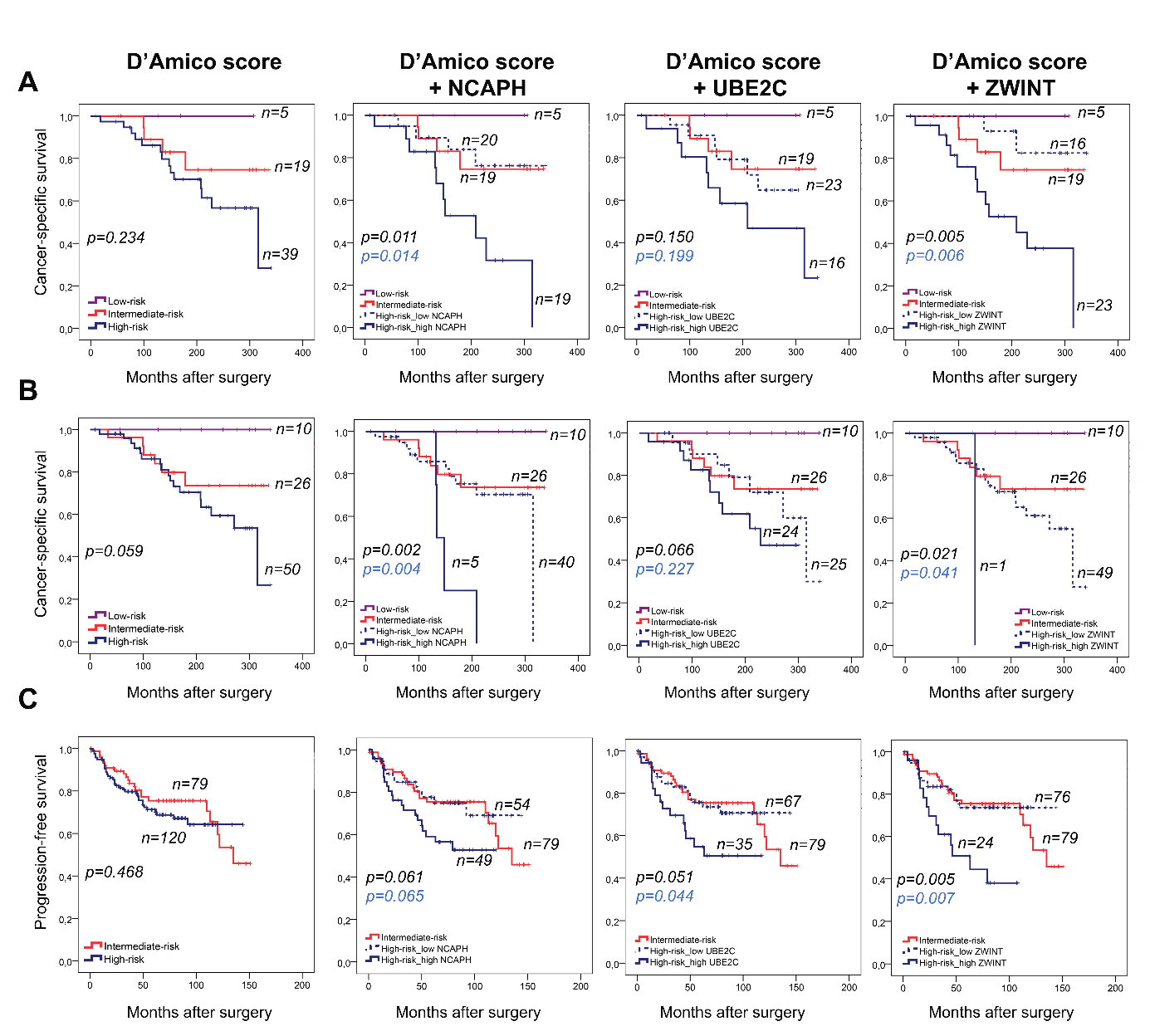


**Supplementary Figure 4**. Cancer-specific survival (CSS) analysis by D’Amico risk scores. High-risk patients were further stratified by the gene (A) and protein (B) expression levels of NCAPH, UBE2C and ZWINT in the institutional GE and IHC cohorts (cohort I and II). Progression-free analyses (PFS) in the multicentre IHC validation cohort (cohort III) stratified by D’Amico risk scores and NCAPH, UBE2C and ZWINT protein expressions (C). P-values represent CSS or PFS differences for all groups (black) and between high-risk patients with low vs. high gene or protein expressions (blue).


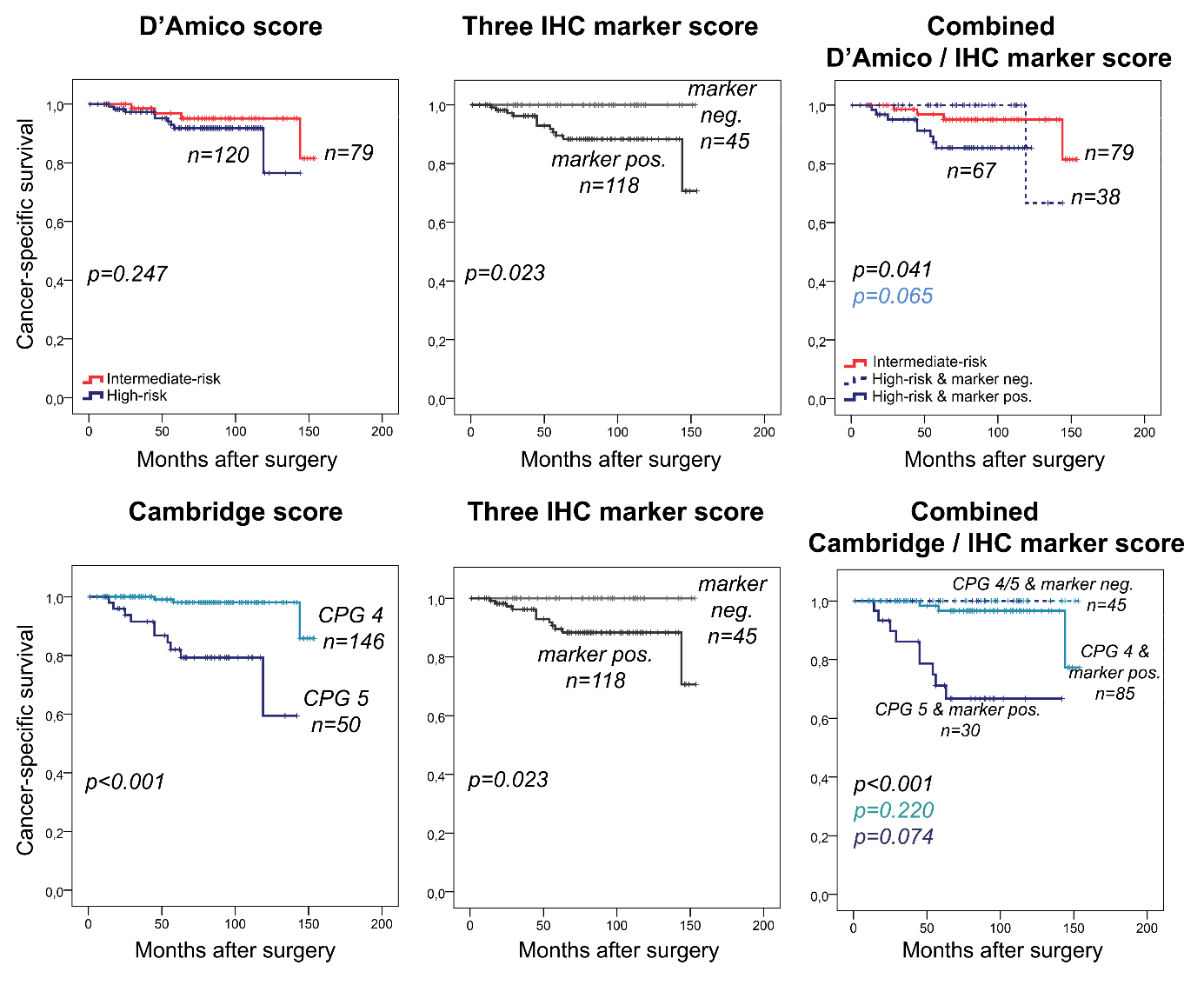


**Supplementary Figure 5.** Cancer-specific survival (CSS) analysis by D’Amico risk and Cambridge scores (left), three IHC marker score (middle), and the combination of D’Amico or Cambridge and IHC marker scores (right) in the multicentre IHC validation cohort (cohort III). D’Amico high-risk and Cambridge prognostic groups (CPG 4 and 5) patients were further stratified by the three marker score (at least one marker positive by IHC). P-values represent CSS differences for all groups (black), and between D’Amico high-risk patients with negative vs. positive protein expressions (blue), and between Cambridge risk group 4 (CPG) patients with negative vs. positive protein expressions (light blue), and between CPG 5 patients with negative vs. positive protein expressions (dark blue). The three IHC marker score evaluation was not feasible in 36 samples due to missing IHC staining intensities of any of the three markers. Neg.: negative marker expression, Pos.: positive expression of any of the three markers.

## *Supplementary Tables*

**Supplementary Table 1.** The 113 prognostic genes identified by *in silico* data analysis. The top 20 genes with the highest average values of hazard ratio (HR) and the lowest p-values based on the TCGA and GSE21034 datasets are marked with blue.

|  | **Probe ID** | **Gene symbol** | **P-value** | **average HR** |
| --- | --- | --- | --- | --- |
| 1 | 209380_s_at | *ABCC5* | 0,001 | 7,941 |
| 2 | 219510_at | *POLQ* | 0,007 | 5,522 |
| 3 | 209464_at | *AURKB* | 0,008 | 6,280 |
| 4 | 207598_x_at | *XRCC2* | 0,009 | 6,827 |
| 5 | 218115_at | *ASF1B* | 0,009 | 6,223 |
| 6 | 219306_at | *KIF15* | 0,010 | 7,754 |
| 7 | 204026_s_at | *ZWINT* | 0,012 | 5,200 |
| 8 | 202240_at | *PLK1* | 0,012 | 5,876 |
| 9 | 206091_at | *MATN3* | 0,012 | 4,596 |
| 10 | 38158_at | *ESPL1* | 0,013 | 4,642 |
| 11 | 210448_s_at | *P2RX5* | 0,013 | 4,743 |
| 12 | 204492_at | *ARHGAP11A* | 0,014 | 7,538 |
| 13 | 219502_at | *NEIL3* | 0,014 | 4,505 |
| 14 | 202954_at | *UBE2C* | 0,014 | 6,409 |
| 15 | 210821_x_at | *CENPA* | 0,015 | 4,971 |
| 16 | 203145_at | *SPAG5* | 0,016 | 5,690 |
| 17 | 206364_at | *KIF14* | 0,016 | 5,276 |
| 18 | 219978_s_at | *NUSAP1* | 0,016 | 4,402 |
| 19 | 212949_at | *NCAPH* | 0,016 | 5,172 |
| 20 | 219472_at | *CENPO* | 0,017 | 4,874 |
| 21 | 202338_at | *TK1* | 0,017 | 5,314 |
| 22 | 203358_s_at | *EZH2* | 0,017 | 5,700 |
| 23 | 206422_at | *GCG* | 0,017 | 4,827 |
| 24 | 204267_x_at | *PKMYT1* | 0,018 | 6,432 |
| 25 | 211519_s_at | *KIF2C* | 0,019 | 5,214 |
| 26 | 218308_at | *TACC3* | 0,020 | 5,570 |
| 27 | 214804_at | *CENPI* | 0,020 | 4,472 |
| 28 | 221520_s_at | *CDCA8* | 0,020 | 5,119 |
| 29 | 208394_x_at | *ESM1* | 0,020 | 7,170 |
| 30 | 218990_s_at | *SPRR3* | 0,021 | 4,616 |
| 31 | 209891_at | *SPC25* | 0,021 | 4,071 |
| 32 | 209773_s_at | *RRM2* | 0,021 | 5,782 |
| 33 | 205046_at | *CENPE* | 0,023 | 4,924 |
| 34 | 214011_s_at | *NOP16* | 0,023 | 4,327 |
| 35 | 204729_s_at | *STX1A* | 0,024 | 5,671 |
| 36 | 207381_at | *ALOX12B* | 0,024 | 3,870 |
| 37 | 216439_at | *TNK2* | 0,024 | 3,995 |
| 38 | 220255_at | *FANCE* | 0,024 | 4,761 |
| 39 | 219990_at | *E2F8* | 0,025 | 4,622 |
| 40 | 204558_at | *RAD54L* | 0,026 | 3,936 |
| 41 | 207862_at | *UPK2* | 0,026 | 3,557 |
|  | **Probe ID** | **Gene symbol** | **P-value** | **average HR** |
| 42 | 215455_at | *TIMELESS* | 0,026 | 5,357 |
| 43 | 40020_at | *CELSR3* | 0,027 | 3,790 |
| 44 | 207192_at | *DNASE1L2* | 0,027 | 4,124 |
| 45 | 219650_at | *ERCC6L* | 0,027 | 4,783 |
| 46 | 204033_at | *TRIP13* | 0,028 | 3,914 |
| 47 | 205913_at | *PLIN1* | 0,029 | 4,875 |
| 48 | 220997_s_at | *DIAPH3* | 0,030 | 4,328 |
| 49 | 207038_at | *SLC16A6* | 0,030 | 3,772 |
| 50 | 208291_s_at | *TH* | 0,030 | 3,600 |
| 51 | 208368_s_at | *BRCA2* | 0,030 | 3,732 |
| 52 | 215550_at | *SRGAP3* | 0,031 | 3,716 |
| 53 | 204827_s_at | *CCNF* | 0,031 | 3,583 |
| 54 | 205064_at | *SPRR1B* | 0,032 | 5,924 |
| 55 | 219769_at | *INCENP* | 0,032 | 4,535 |
| 56 | 219403_s_at | *HPSE* | 0,033 | 4,730 |
| 57 | 207669_at | *KRT83* | 0,033 | 5,572 |
| 58 | 201292_at | *TOP2A* | 0,034 | 4,243 |
| 59 | 218355_at | *KIF4A* | 0,034 | 4,721 |
| 60 | 220668_s_at | *DNMT3B* | 0,034 | 4,580 |
| 61 | 219621_at | *CLSPN* | 0,035 | 3,763 |
| 62 | 210503_at | *MAGEA11* | 0,036 | 3,591 |
| 63 | 211619_s_at | *ALPP* | 0,036 | 3,376 |
| 64 | 221965_at | *MPHOSPH9* | 0,037 | 4,093 |
| 65 | 221572_s_at | *SLC26A6* | 0,037 | 4,818 |
| 66 | 210040_at | *SLC12A5* | 0,037 | 4,050 |
| 67 | 212023_s_at | *MKI67* | 0,038 | 4,024 |
| 68 | 211320_s_at | *PTPRU* | 0,038 | 3,712 |
| 69 | 216422_at | *PA2G4* | 0,038 | 3,453 |
| 70 | 208102_s_at | *PSD* | 0,038 | 3,342 |
| 71 | 214383_x_at | *KLHDC3* | 0,039 | 5,073 |
| 72 | 206177_s_at | *ARG1* | 0,039 | 3,401 |
| 73 | 202779_s_at | *UBE2S* | 0,039 | 3,605 |
| 74 | 216663_s_at | *ZMYND10* | 0,039 | 4,281 |
| 75 | 210334_x_at | *BIRC5* | 0,039 | 3,232 |
| 76 | 219918_s_at | *ASPM* | 0,040 | 4,283 |
| 77 | 209159_s_at | *NDRG4* | 0,040 | 3,843 |
| 78 | 209875_s_at | *SPP1* | 0,040 | 5,193 |
| 79 | 202870_s_at | *CDC20* | 0,041 | 5,024 |
| 80 | 216302_at | *HNRNPC* | 0,041 | 4,528 |
| 81 | 211214_s_at | *DAPK1* | 0,041 | 5,673 |
| 82 | 205733_at | *BLM* | 0,041 | 5,655 |
| 83 | 213171_s_at | *MMP24* | 0,042 | 3,912 |
| 84 | 213801_x_at | *RPSA* | 0,042 | 3,838 |
| 85 | 219753_at | *STAG3* | 0,042 | 4,529 |
| 86 | 219785_s_at | *FBXO31* | 0,042 | 3,694 |
| 87 | 216038_x_at | *DAXX* | 0,043 | 4,289 |
|  | **Probe ID** | **Gene symbol** | **P-value** | **average HR** |
| 88 | 203968_s_at | *CDC6* | 0,043 | 4,127 |
| 89 | 214347_s_at | *DDC* | 0,043 | 5,513 |
| 90 | 214969_at | *MAP3K9* | 0,044 | 6,426 |
| 91 | 220625_s_at | *ELF5* | 0,044 | 3,458 |
| 92 | 217305_s_at | *ADCY10* | 0,045 | 5,300 |
| 93 | 220269_at | *ZBBX* | 0,045 | 4,419 |
| 94 | 220733_at | *SLC26A1* | 0,046 | 3,150 |
| 95 | 212477_at | *ACAP2* | 0,046 | 4,712 |
| 96 | 221990_at | *PAX8* | 0,046 | 3,215 |
| 97 | 220344_at | *C11orf16* | 0,046 | 3,374 |
| 98 | 206134_at | *ADAMDEC1* | 0,046 | 3,951 |
| 99 | 222353_at | *LIMD1* | 0,047 | 4,926 |
| 100 | 216513_at | *DCT* | 0,047 | 3,529 |
| 101 | 210117_at | *SPAG1* | 0,047 | 6,926 |
| 102 | 214079_at | *DHRS2* | 0,047 | 3,339 |
| 103 | 215047_at | *TRIM58* | 0,047 | 5,614 |
| 104 | 202022_at | *ALDOC* | 0,048 | 4,164 |
| 105 | 208036_at | *OPN1SW* | 0,048 | 3,298 |
| 106 | 205635_at | *KALRN* | 0,048 | 3,820 |
| 107 | 203362_s_at | *MAD2L1* | 0,048 | 3,972 |
| 108 | 218755_at | *KIF20A* | 0,049 | 4,278 |
| 109 | 210106_at | *RDH5* | 0,049 | 3,649 |
| 110 | 216835_s_at | *DOK1* | 0,049 | 4,604 |
| 111 | 205676_at | *CYP27B1* | 0,050 | 3,392 |
| 112 | 207226_at | *HIST1H2BN* | 0,050 | 3,214 |
| 113 | 220791_x_at | *SCN11A* | 0,050 | 4,630 |

**Supplementary Table 2.** Wilcoxon rank-sum test between patients’ clinicopathological parameters and continuous gene expression values in the institutional GE cohort (cohort I). The table includes only those markers that showed significant association with at least one clinicopathological parameter. Markers without significant associations with any of the clinicopathological parameters are not included. KW: Kruskal-Wallis test was performed for the continuous clinical parameters (variables). Med: median gene expression. Bold printed p-values were significant (≤0.05).

| **Variables** | ***ASF1B*** | | | ***AURKB*** | | | ***CENPA*** | | | ***ESPL1*** | | | ***NUSAP1*** | | | ***P2RX5*** | | | ***UBE2C*** | | |
| --- | --- | --- | --- | --- | --- | --- | --- | --- | --- | --- | --- | --- | --- | --- | --- | --- | --- | --- | --- | --- | --- |
|  | med | range | P | med | range | P | med | range | P | med | range | P | med | range | P | med | range | P | med | range | P |
| Age |  |  |  |  |  |  |  |  |  |  |  |  |  |  |  |  |  |  |  |  |  |
| ≤ 65 | 18 | 2-150 | 0.762 | 21 | 1-272 | 0.213 | 26 | 2-150 | 0.522 | 8.5 | 1-154 | 0.646 | 25.5 | 2-241 | 0.894 | 43 | 8-1458 | 0.523 | 36 | 3-226 | 0.452 |
| > 65 | 19 | 4-69 |  | 15 | 5-164 |  | 22 | 2-108 |  | 7 | 1-90 |  | 22 | 5-103 |  | 49 | 17-467 |  | 34 | 5-246 |  |
| Pathological stage |  |  |  |  |  |  |  |  |  |  |  |  |  |  |  |  |  |  |  |  |  |
| T1-pT2 | 17 | 2-72 | 0.156 | 22 | 5-193 | 0.236 | 26 | 3-150 | 0.363 | 12 | 1-154 | **0.046** | 18 | 2-57 | **0.035** | 74 | 11-467 | **0.030** | 37 | 5-137 | 0.880 |
| pT3-pT4 | 21.5 | 3-150 |  | 16 | 1-272 |  | 22.5 | 2-92 |  | 3 | 1-92 |  | 33 | 3-241 |  | 35 | 8-1458 |  | 35 | 3-226 |  |
| Lymph node status |  |  |  |  |  |  |  |  |  |  |  |  |  |  |  |  |  |  |  |  |  |
| LN0 | 18 | 2-92 | 0.237 | 19 | 1-193 | 0.056 | 23 | 2-150 | 0.476 | 6.5 | 1-154 | 0.908 | 23.5 | 2-103 | 0.309 | 48.5 | 8-1548 | 0.174 | 34 | 3-246 | **0.022** |
| LN+ | 29 | 10-150 |  | 28 | 17-272 |  | 28 | 10-59 |  | 12 | 1-43 |  | 31 | 17-241 |  | 24 | 15-467 |  | 73 | 49-229 |  |
| PSA |  |  |  |  |  |  |  |  |  |  |  |  |  |  |  |  |  |  |  |  |  |
| < 10 ng/ml | 14 | 1-72 | 0.125 | 16 | 5-162 | 0.203 | 13 | 2-150 | **0.026** | 8.5 | 1-154 | 0.766 | 19.5 | 2-86 | 0.087 | 73.5 | 11-567 | **0.018** | 28 | 5-134 | 0.065 |
| ≥ 10 ng/ml | 23 | 3-150 |  | 20.5 | 1-272 |  | 26 | 3-108 |  | 6.5 | 1-92 |  | 29.5 | 3-241 |  | 31.5 | 8-1548 |  | 38 | 3-246 |  |
| PSA as cont. var. (KW) | 18 | 1-150 | 0.469 | 20 | 1-272 | 0.630 | 25 | 2-150 | 0.532 | 7 | 1-154 | 0.483 | 24 | 2-241 | 0.564 | 46 | 8-1458 | 0.506 | 36 | 3-246 | 0.424 |
| ISUP/WHO grade group |  |  |  |  |  |  |  |  |  |  |  |  |  |  |  |  |  |  |  |  |  |
| 1 | 14 | 1-69 | 0.052 | 15 | 5-193 | 0.371 | 21 | 2-150 | 0.251 | 14 | 1-154 | **0.044** | 16 | 2-103 | **0.001** | 74 | 8-467 | **0.021** | 26 | 5-246 | **0.013** |
| 2-5 | 24.5 | 3-150 |  | 20.5 | 1-272 |  | 26 | 2-108 |  | 3 | 1-92 |  | 34 | 3-241 |  | 30.5 | 11-1458 |  | 47 | 3-226 |  |
| ISUP continuous (KW) | 18 | 1-150 | 0.057 | 20 | 1-272 | 0.100 | 25 | 2-150 | 0.053 | 7 | 1-154 | 0.150 | 24 | 2-241 | **0.004** | 46 | 8-1458 | 0.140 | 36 | 3-246 | 0.073 |
| D'Amico |  |  |  |  |  |  |  |  |  |  |  |  |  |  |  |  |  |  |  |  |  |
| risk group 1-2 | 17.5 | 2-72 | **0.044** | 15 | 1-164 | **0.044** | 13 | 2-91 | **0.006** | 12.5 | 1-90 | 0.943 | 22 | 2-57 | 0.147 | 80 | 11-467 | 0.168 | 32 | 3-137 | 0.232 |
| risk group 3 | 25 | 4-150 |  | 22 | 6-272 |  | 27 | 2-150 |  | 6 | 1-154 |  | 29 | 2-241 |  | 37 | 8-1458 |  | 36 | 8-246 |  |
| D'Amico continuous (KW) | 18 | 2-150 | 0.116 | 20 | 1-272 | 0.127 | 25 | 2-150 | **0.021** | 7 | 1-154 | 0.216 | 24 | 2-241 | 0.349 | 46 | 8-1458 | 0.110 | 36 | 3-246 | 0.458 |
| CAPRA |  |  |  |  |  |  |  |  |  |  |  |  |  |  |  |  |  |  |  |  |  |
| risk group 1-2 | 15.5 | 2-92 | **0.005** | 15 | 1-164 | **0.036** | 19 | 2-150 | **0.042** | 11 | 1-154 | 0.214 | 22.5 | 2-103 | **0.026** | 72 | 11-1458 | **0.014** | 31.5 | 3-246 | **0.036** |
| risk group 3 | 30.5 | 8-150 |  | 24.5 | 10-272 |  | 28.5 | 2-108 |  | 3.5 | 1-56 |  | 33 | 13-241 |  | 30.5 | 8-207 |  | 49.5 | 14-226 |  |
| CAPRA continuous (KW) | 18 | 2-150 | **0.020** | 20 | 1-272 | 0.085 | 25 | 2-150 | 0.119 | 7 | 1-154 | 0.160 | 24 | 2-241 | **0.050** | 46 | 8-1458 | **0.044** | 36 | 3-246 | 0.066 |

**Supplementary Table 3.** Cox uni – (A) and multivariate (B) analysis in the institutional GE (cohort I) and IHC cohorts (cohorts II and III). GE: gene expression. HR: hazard ratio, CI: confidence interval, Ref: referent, na: not available data, nf: no feasible evaluation. Bold printed p-values were significant (≤0.05).

**Supplementary Table 4.** Combined IHC evaluation of NCPAH, UBE2C, and ZWINT. Bold printed p-values were significant (≤0.05).

| **A** | **Cancer-specific survival** | | | |
| --- | --- | --- | --- | --- |
| *Institutional IHC cohort (cohort II)* | n | HR | 95% CI | P |
| Variables |  |  |  |  |
| High IHC score for minimum 1 marker | 43 | 5.641 | 1.640-19.406 | **0.006** |
| High IHC score for minimum 2 marker | 9 | 3.218 | 1.245-8.321 | **0.016** |
| High IHC score for all the 3 markers | 1 | 9.109 | 1.138-72.880 | **0.037** |
| **B** | **Cancer-specific survival** | | | |
| *Multicentre IHC validation cohort (cohort III)* | n | HR | 95% CI | P |
| Variables |  |  |  |  |
| High IHC score of min 1 marker | 118 | 6.249 | 0.812-48.073 | 0.078 |
| High IHC score of min 2 marker | 69 | 2.623 | 0.858-8.106 | 0.091 |
| High IHC score of each marker | 14 | 3.887 | 1.051-14.377 | **0.042** |
| **C** | **Progression-free survival** | | | |
| *Multicentre IHC validation cohort (cohort III)* | n | HR | 95% CI | P |
| Variables |  |  |  |  |
| High IHC score for minimum 1 marker | 118 | 2.399 | 1.126-5.113 | **0.023** |
| High IHC score for minimum 2 marker | 69 | 1.857 | 1.066-3.234 | **0.029** |
| High IHC score for all the 3 markers | 14 | 2.703 | 1.311-5.572 | **0.007** |

## *References*

1. Castiglione F, Dell’Oglio P, Tosco L, Everaerts W, Albersen M, Hakim L, et al. Tumor Volume and Clinical Failure in High-Risk Prostate Cancer Patients Treated With Radical Prostatectomy. Prostate **77**(1), 3–9 (2017).
2. 6. D’Amico A V, Whittington R, Bruce Malkowicz S, Schultz D, Blank K, Broderick GA, et al. Biochemical outcome after radical prostatectomy, external beam radiation therapy, or interstitial radiation therapy for clinically localized prostate cancer. J Am Med Assoc **280**(11), 969–974 (1998).
3. Gnanapragasam VJ, Bratt O, Muir K, Lee LS, Huang HH, Stattin P, Lophatananon A. The Cambridge Prognostic Groups for improved prediction of disease mortality at diagnosis in primary non-metastatic prostate cancer: A validation study. BMC Med **16**(1), 31 (2018).
4. Cooperberg M, Pasta D, Elkin E, Litwin M, Latini DM, Du Chane J, Carroll PR. The UCSF Cancer of the Prostate Risk Assessment (CAPRA) Score: a straightforward and reliable preoperative predictor of disease recurrence after radical prostatectomy. J Urol **173**(6), 1938–1942 (2005).
